# Classification of caesarean section and normal vaginal deliveries using foetal heart rate signals and advanced machine learning algorithms

**DOI:** 10.1186/s12938-017-0378-z

**Published:** 2017-07-06

**Authors:** Paul Fergus, Abir Hussain, Dhiya Al-Jumeily, De-Shuang Huang, Nizar Bouguila

**Affiliations:** 1 Applied Computing Research Group, Department of Computer Science, Faculty of Engineering and Technology, Liverpool John Moors University, Byron Street, Liverpool, L3 3AF UK; 2 Institute of Machine Learning and Systems Biology, Tongji University, No. 4800 Caoan Road, Shanghai, 201804 China; 3 Concordia Institute for Information Systems Engineering, Concorida University, 1455 de Maisonneuve Blvd West, EV7.632, Montreal, QC HJ3G 2W1 Canada

**Keywords:** Classification, Feature extraction and selection, Deep learning, Intrapartum cardiotocography, Machine learning, Random forest

## Abstract

**Background:**

Visual inspection of cardiotocography traces by obstetricians and midwives is the gold standard for monitoring the wellbeing of the foetus during antenatal care. However, inter- and intra-observer variability is high with only a 30% positive predictive value for the classification of pathological outcomes. This has a significant negative impact on the perinatal foetus and often results in cardio-pulmonary arrest, brain and vital organ damage, cerebral palsy, hearing, visual and cognitive defects and in severe cases, death. This paper shows that using machine learning and foetal heart rate signals provides direct information about the foetal state and helps to filter the subjective opinions of medical practitioners when used as a decision support tool. The primary aim is to provide a proof-of-concept that demonstrates how machine learning can be used to objectively determine when medical intervention, such as caesarean section, is required and help avoid preventable perinatal deaths.

**Methods:**

This is evidenced using an open dataset that comprises 506 controls (normal virginal deliveries) and 46 cases (caesarean due to pH ≤ 7.20—acidosis, n = 18; pH > 7.20 and pH < 7.25—foetal deterioration, n = 4; or clinical decision without evidence of pathological outcome measures, n = 24). Several machine-learning algorithms are trained, and validated, using binary classifier performance measures.

**Results:**

The findings show that deep learning classification achieves sensitivity = 94%, specificity = 91%, Area under the curve = 99%, F-score = 100%, and mean square error = 1%.

**Conclusions:**

The results demonstrate that machine learning significantly improves the efficiency for the detection of caesarean section and normal vaginal deliveries using foetal heart rate signals compared with obstetrician and midwife predictions and systems reported in previous studies.

## Introduction

Worldwide, over 130 million babies are born each year. 3.6 million will die due to perinatal complication and 1 million of these will be intrapartum still births [1]. In the USA, the number of deliveries in 2012 was 3952,841; one in every 164 of these resulted in stillbirth.^1^ In the UK, in the same year, there were 671,255 with one in every 200 being stillbirth^2^ and 300 that died in the first four weeks of life [2].

Cardiotocography (CTG) is the most common method used to monitor the foetus during the early stages of delivery [3] and clinical decisions are made using the visual inspection of CTG traces. However, the main weakness with this approach is poor human interpretation which leads to high inter- and intra-observer variability [4]. While significant pathological outcomes like hypoxia are uncommon, false alarms are not, which can lead to serious abnormalities, such as cardio-pulmonary arrest, brain and vital organ damage, cerebral palsy, hearing, visual and cognitive defects and in severe cases, death, being overlooked [5]. Conversely, over interpretation of CTG is common and the direct cause of unnecessary caesarean sections (CS). In such cases, between 40 and 60% of babies are born without any evidence to support pathological outcomes, such as hypoxia and metabolic acidosis [6].

This paper aims to address this problem by incorporating a proof-of-concept system alongside existing gold standard methods in antenatal care. Using foetal heart rate signals and machine learning an objective measure of foetal state is used to detect the onset of pathological cases. This will provide obstetricians and midwives with an additional level of foetal state interpretation and help decide if and when surgical intervention is required. The results show that the approach has superior predictive capacity when compared with the 30% positive predictive value produced by obstetricians and midwives when classifying normal vaginal and caesarean section deliveries [7].

The remainder of this paper is organized as follows: “Background” provides a brief description of CTG as a screening tool for foetal hypoxia and its causes, and the findings of related work. “Methods” describes the dataset adopted in this paper and various steps involved. “Results” presents the classification outcomes for both control and case records, and the findings are discussed in “Discussion”. The paper is concluded in “Conclusions and future work”.

## Background

CTG was initially developed as a screening tool to predict foetal hypoxia [7]. However, there is no evidence to suggest that there has been any improvement in perinatal deaths since the introduction of CTG into clinical practice 45 years ago. It is generally agreed that 50% of birth-related brain injuries are preventable, with incorrect CTG interpretation leading the list of causes [8]. Equally, over interpretation of CTG is common and the direct cause of unnecessary caesarean sections, which costs the NHS £1700 for each caesarean performed compared with £750 for a normal vaginal delivery. It is therefore generally agreed that predicting adverse pathological outcomes and diagnosing pathological outcomes earlier clearly have important consequences, for both health and the economy. One interesting approach is machine learning.

Warrick et al. [9] developed a system for the classification of normal and hypoxic foetuses by modelling the FHR and uterine contraction (UC) signal pairs as an input–output system to estimate their dynamic relation in terms of an impulse response function [10]. The authors report that their system can detect almost half of the pathological cases 1 h and 40 min prior to delivery with a 7.5% false positive rate. Kessler et al. [11] on the other hand, using 6010 high risk deliveries, combined CTG with ST waveform to apply timely intervention for caesarean or vaginal delivery, which they report, reduced foetal morbidity and mortality [12].

In comparative studies, Huang et al. [13] compared three different classifiers; a decision tree (DT), an artificial neural network (ANN), and discriminant analysis (DA). Using the ANN classifier, it was possible to obtain a 97.78% overall accuracy. This was followed by the DT and DA with 86.36 and 82.1% accuracy respectively. The sensitivity and specificity values were not provided making accuracy alone an insufficient performance measure for binary classifiers. This is particularly true in evaluations where datasets are skewed in favour of one class with significant differences between prior probabilities.

In a similar study, Ocak et al. [14] evaluated an SVM and genetic algorithm (GA) classifier and reported 99.3 and 100% accuracies for normal and pathological cases respectively. Similar results were reported in [15, 16]. Again, Sensitivity and Specificity values where not provided in these studies. Meanwhile Menai et al. [17] carried out a study to classify foetal state using a naive bayes (NB) classifier with four different feature selection (FS) techniques: Mutual information, correlation-based, ReliefF, and information gain. The study found that the NB classifier in conjunction with features produced using the ReliefF technique produce the best results when classifying foetal state with 93.97, 91.58, and 95.79% for accuracy, sensitivity and specificity, respectively. While the results are high, the dataset is multivariate and highly imbalanced. Alternative model evaluation metrics for multi-class data, such as micro- and macro-averaging, and micro and macro-F-measure, would provide a more informed account of model performance. Furthermore, an appropriate account of how the class skew problem was addressed is missing.

The adaptive boosting (AdaBoost) classifier was adopted in a study by Karabulut et al. [18] who report an accuracy of 95.01%—again no sensivity or specificity values were provided. While Spilka et al. who are the current forerunners of pioneering work in machine learning and CTG classification [6], used a random forest (RF) classifier in conjunction with latent class analysis (LCA) [19] and reported sensitivity and specificity values of 72 and 78% respectively using the CTG-UHB dataset [3]. Producing slightly better results in [20] using the same dataset, Spilka et al. attempted to detect hypoxia using a C4.5 decision tree, naive bayes, and SVM. The SVM produced the best results using a tenfold cross validation method achieving 73.4% for sensitivity and 76.3% of specificity.

## Methods

This section describes the dataset adopted in this study and discusses the steps taken to pre-process the data and extract the features from raw FHR signals. The section is then concluded with a discussion on the feature selection technique and dimensionality reduction.

### CTG data collection

Chudacek et al. [3] conducted a comprehensive study that captured intrapartum recordings between April 2010 and August 2012. The recordings were collected from the University Hospital in Brno (UHB), in the Czech Republic by obstetricians with the support of the Czech Technical University (CTU) in Prague. These records are publically available from the CTU-UHB database, in Physionet [3].

**Fig. 1:**
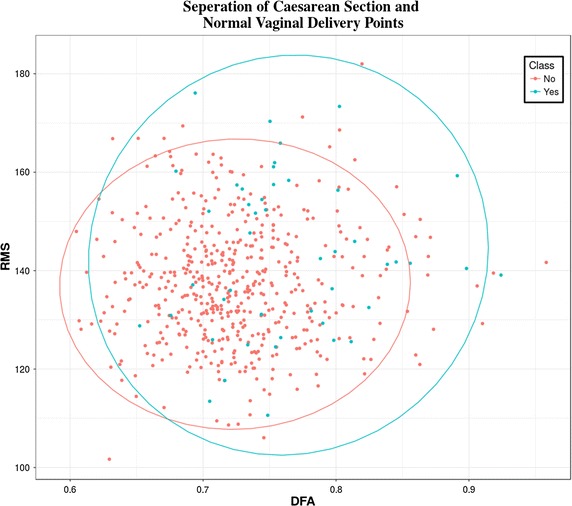
Separation of caesarean section and normal vaginal delivery points

The CTU-UHB database contains 552 CTG recordings for singleton pregnancies with a gestational age less than 36 weeks that were selected from 9164 recordings. The STAN S21/S31 and Avalon FM 40/50 foetal monitors were used to acquire the CTG records. The dataset contains no prior known development factors (i.e. they are ordinary clean obstetrics cases); the duration of stage two labour is less than or equal to 30 min; foetal heart rate signal quality is greater than 50% in each 30 min’ window; and the pH umbilical arterial blood sample is available. In the dataset, 46 caesarean section deliveries are included and the rest are ordinary clean vaginal deliveries. Figure 1 shows a scatter plot of the dataset with eclipses defining the separation between both case and control groups. Note that in this study 46 cases are classified as a caesarean delivery due to pH ≤7.20—acidosis, n = 18; pH >7.20 and pH ≤7.25—foetal deterioration, n = 4; or clinical decision without evidence of pathological outcome measures, n = 24 as defined in [14]. Table 1 provides details of the outcome measures used in the CTU-UHB database.

**Table 1:** Caesarean section outcome measures

ID	Age	pH	BDecf	pCO_2_	BE	Apgar1	Apgar5	Dev type
2001	30	7.03	22.52	2.8	−23.7	10	10	2
2002	39	7.27	3.75	6.5	−4.5	7	4	2
2003	25	6.96	16.96	7.2	−19	6	8	2
2004	34	6.95	11.44	11.6	−15.3	6	8	2
2005	31	7.25	3.47	7	−5.5	10	10	2
2006	32	7.29	NaN	NaN	NaN	10	10	2
2007	27	7.04	20.42	3.8	−21.8	10	10	2
2008	26	6.98	13.43	9.3	−16.7	5	7	2
2009	21	6.96	20.34	5.4	−23	10	10	2
2010	19	7.3	−0.48	7.2	−1.5	10	10	2
2011	37	7.01	12.1	9.2	−14.8	3	7	2
2012	26	7.29	−0.44	7.4	−1.4	9	9	2
2013	27	6.85	22.63	6.4	−25.3	8	8	2
2014	34	7.32	2.28	6	−3.2	10	10	2
2015	29	7.33	4.15	5.3	−5.1	9	10	2
2016	38	7.27	1.88	7.1	−3.8	9	10	2
2017	34	7.32	−0.16	6.7	−2	10	10	2
2018	30	7.31	3.93	5.7	−5	10	10	2
2019	31	7.29	4.13	6	−5.6	9	9	2
2020	28	7.15	3.09	9.6	−5.8	4	7	2
2021	28	7.3	0.19	7	−2.2	9	10	2
2022	31	7.28	−0.38	7.6	−1.6	9	10	2
2023	28	6.98	14.49	8.7	−17.4	6	8	2
2024	39	7.01	7.14	12.1	−10.9	2	4	2
2025	29	6.99	12.61	9.5	−16	8	8	2
2026	32	7.23	−0.13	8.7	−2.1	10	10	2
2027	26	7.31	1.88	6.3	−3.2	9	10	2
2028	36	7.18	4.82	8.1	−7.2	8	9	2
2029	34	7.28	1.22	7.1	−3.4	10	10	2
2030	42	7.04	26.11	0.7	−26.8	10	10	2
2031	26	7.29	1.52	6.8	−2.9	9	9	2
2032	35	7.26	3.14	6.9	−4.7	9	10	2
2033	26	7.39	0.86	5.2	−1.5	9	9	2
2034	34	7.34	NaN	NaN	NaN	9	9	2
2035	27	7.26	2.23	7.2	−4.3	8	9	2
2036	34	7.29	2.5	6.5	−3.7	5	7	2
2037	29	7.25	1.09	7.8	−3	9	10	2
2038	27	7.36	3.5	5	−4	5	8	2
2039	29	7.32	−0.51	6.8	−0.5	9	10	2
2040	23	7.23	5.27	6.8	−7	2	6	2
2041	32	7.37	3.69	4.8	−3.1	9	9	2
2042	27	7.33	−0.5	6.6	−0.8	9	10	2
2043	26	7.08	10.92	7.9	−13.3	8	9	2
2044	27	7.02	9.13	10.6	−12.3	8	8	2
2045	32	7.03	8.91	10.4	−12.2	7	9	2
2046	19	7.01	NaN	NaN	NaN	5	7	2

The ID column in Table 1 maps to the file number in the CTU-UHB database, while the second column provides details about the mothers age. The pH column describes the umbilical artery pH value for each case and BDef provides information on the base deficit in extracellular fluid. The PCO_2_ describes the partial pressure of carbon dioxide. BE gives values for the base excess and finally Apgar scores are a subjective evaluation of the outcome of the delivery. For a more in-depth discussion of the dataset and these parameters please refer to [3].

Each recording begins no more than 90 min before delivery. Each CTG record contains the FHR time series (measured in beats per minute) and uterine contraction (UC) signal—each sampled at 4 Hz. The FHR was obtained from an ultrasound transducer attached to the abdominal wall. In this study only the FHR signal is only considered in this study since it provides direct information about the foetal state.

### Pre-processing

Each of the 552 FHR signal recordings were filtered using a 6th order low-pass butterworth filter with fc = 4 Hz and a cut-off frequency of 0.034 Hz. To correct the phase distortion introduced by a one-pass filter, a two-pass filter (forwards and reverse) was used to filter each of the signals. Noise, and missing values were removed using cubic Hermite spline interpolation [21].

### FHR features

This section describes the statistical, higher-order statistical and higher-order spectral features extracted from the FHR signals.

#### Morphological features

The initial set of features considered are those defined by the international federation of gynecology and obstetrics^3^ (FIGO) and the National Institute for Health and Care Excellence^4^ (NICE). Consider a raw FHR time series signal X with length N, where X = {x_n_, n = 1, 2…, N}, in which the virtual baseline mean (VBM), $\overline{x}$ is defined as:

1 $$ {\bar{\text{x}}} = \frac{{\mathop \sum \nolimits_{n = 1}^{N} x_{n} }}{\text{N}} $$

Such that $\overline{\text{x}}$ can be used to remove accelerations and decelerations ($if\,x_{n} > 10 + \overline{\text{x}} \;{\text{then}}{:}\,x_{n} = {\bar{\text{x}}} + 10;\;if\,x_{n} < - 10 + \overline{\text{x}} \;{\text{then}}{:}\, x_{n} = {\bar{\text{x}}} + - 10$ from the FHR signal so that the real baseline FHR (RBL) can be derived [22]:

2 $$ {\text{RBL}} = \frac{{\int\nolimits_{\text{L}}^{\text{H}} {\text{X}} }}{\text{N}} $$

where *H* and *L* are the upper and lower limits of the time series signal respectively, *X* is the signal and *N* is the length of the signal.

Using the RBL, FIGO accelerations and decelerations can be extracted. These are features commonly used by obstetricians to monitor the interplay between the sympathetic and parasympathetic systems. Accelerations and decelerations within *X* represent the transient increases and decreases (±15 bpm) that last for 15 s or more [23]. In the case of accelerations, this typically indicates adequate blood delivery and is reassuring for the obstetrician. Calculating accelerations in the signal is defined by:

3 $$ Acc_{total} = \exists x_{i} \in X, x_{i} \ge RBL + 15 \;\& \; D \ge 15 $$

where *X* is the signal, $x_{i}$ is the *i*th element of *X*, RBL is the real baseline defined in (2), and *D* is the duration of time in which *x*_*i*_ remains above RBL +15.

In contrast decelerations represent temporary decreases (−15 bpm) in FHR below the RBL that last for 15 s or more, which can indicate the presence of possible pathological outcomes such as, umbilical cord compression, hypoxia or acidosis [18]. The decelerations in the signal are calculated as:

4 $$ Dec_{total} = \exists x_{i} \in X, x_{i} \le RBL - 15 \;\& \; D \ge 15 $$

where $x_{i}$ is the *i*th element of signal *X*, RBL is the real baseline, and *D* is the time duration in which *x*_*i*_ remains below RBL-15.

Short and long-term variability (STV and LTV respectively) are further indicators used by obstetricians. The presence of both suggests an intact neuromodulation of the FHR and normal cardiac function and is one of the most reassuring measures in neonatal care [24]. When STV or LTV decreases or is absent, it can be a significant indicator for the presence of hypoxia or acidosis. Therefore, they are both considered to be important predictors. STV is calculated according to the following equation:

5 $$ STV = \frac{1}{M}\mathop \sum \limits_{t = 1}^{M} R_{t} $$

where *M* is the number of minutes contained in the *X* signal and $R_{t}$ is defined as:

6 $$ R_{t} = \frac{1}{H - 1}\mathop \sum \limits_{j}^{H - 1} \left| {\overline{S}_{j} - \overline{S}_{j + 1} } \right| $$

where *H* is the number of subintervals in 60 s (in this case H = 60/K), *K* is the sample frequency (4 Hz) multiplied by 2.5 s and $\overline{S}_{j}$ is the average value of 2.5 s for a subinterval *j* = {1, 2,…,H}.

In contrast, LTV is defined as the difference between the minimum and maximum value in a 60-s block and is averaged to the duration of the signal if it is more than 1 min long. LTV is defined as:

7 $$ LTV = \frac{1}{N/60}\mathop \sum \limits_{i = 1}^{N} \left[ {\mathop {\hbox{max} }\limits_{i \in N} \left( {X\left( {i + b} \right)} \right) - \mathop {\hbox{min} }\limits_{i \in M} \left( {X\left( {i + b} \right)} \right)} \right] $$

where *N* is the length of the *X* signal, *b* is 240 samples (60-s blocks for a 4 Hz sample frequency).

Collectively, RBL, accelerations, decelerations, STV and LTV define the five main FIGO/NICE features used by obstetricians and midwives and are subsequently consider as predictors for separating caesarean section and normal vaginal deliveries in this study.

#### Time series features

FIGO feature sets are often extended in automated CTG analysis to include patterns in the signal that are not easily identifiable through visual inspection. Two useful time-series features that have been heavily utilized in medical signal processing are root mean squares (RMS) and sample entropy (SampEn). RMS is a useful feature for estimating short term variability between accelerations and deceleration [25] and is commonly described for a signal *X* with length *N* as:

8 $$ RMS = \sqrt {\frac{1}{N}\mathop \sum \limits_{i = 0}^{N - 1} x_{i}^{2} } $$

This feature is particularly good at estimating sympathetic/parasympathetic dominance where the later, in a similar way to decelerations, can indicate the presence of possible pathological incidences, such as hypoxia and acidosis.

Whereas, sample entropy, quantifies the nonlinear dynamics of the FHR and the loss of complexity in the FHR signal. Previous studies have reported that it is a worthwhile feature for determining if the foetus is deprived of oxygen [26]. Sample entropy is the negative natural logarithm of the conditional probability that a dataset of length *N*, having repeated itself for *m* samples within a tolerance of *r*, will also repeat itself for *m* + 1 samples. Based on the calculation in [27] the time series *X* that contains *N* points, $x_{i} ,\;x_{2} \ldots ,\;x_{N}$ subsequences can be defined by length *m*, and given by: $y_{i} = (x_{i} , \;x_{i + 1} , \ldots .,x_{i + m - 1} )$ where i = 1, 2, …, *N* – *m* + 1. This allows the following quantity to be defined: $B_{i}^{m} \left( r \right)$ as $(N - m - 1)^{ - 1}$ times the number of vectors $V_{j}^{m}$ within r of $V_{i}^{m}$, where *j* ranges from 1 to *N*−*m*, and $j \ne i$, to exclude self-matches, followed by:

9 $$ B^{m} \left( r \right) = \frac{1}{n - m}\mathop \sum \limits_{i = 1}^{N - m} B_{i}^{m} (r) $$

Similarly, $A_{i}^{m} \left( r \right)$ is defined as $(N - m - 1)^{ - 1}$ times the number of vectors $V_{j}^{m + 1}$ within *r* of $V_{i}^{m + 1}$, where *j* ranges from 1 to *N*−*m*, and $j \ne i$, and set:

10 $$ A^{m} \left( r \right) = \frac{1}{n - m}\mathop \sum \limits_{i = 1}^{N - m} A_{i}^{m} (r) $$

The parameter SampEn(*m*, *r*) is then defined as:

11 $$ \lim_{N \to \infty } \left\{ { - \ln \left[ {\frac{{A^{m} \left( r \right)}}{{B^{m} \left( r \right)}}} \right]} \right\} $$

Which can be estimated by the statistic:

12 $$ SampEn\left( {m,r, N} \right) = \left\{ { - \ln \left[ {\frac{{A^{m} \left( r \right)}}{{B^{m} \left( r \right)}}} \right]} \right\} $$

where *N* is the length of the *X* signal, *m* is the length of sequences to be compared, and *r* is the tolerance for accepted matches.

#### Frequency domain features

To overcome signal quality variations in the FHR signal, due to electrode placement and the physical characteristics of subjects [28], frequency domain features have been studied using power spectral density (PSD) computed using fast fourier transform (FFT):

13 $$ {\mathbf{X}}\left( {\mathbf{f}} \right) = \int\nolimits_{{ - \infty }}^{{ + \infty }} {{\mathbf{x}}\left( {\mathbf{t}} \right){\mathbf{e}}^{{ - {\mathbf{j}}2{\mathbf{\pi ft}}}} {\mathbf{dt}}~\;{\mathbf{and}} - \infty < f < + \infty } $$

where X(*f*) contains the information for the signal and x(*t*) is obtained from X(*f*) using the inverse of the fourier transformation:

14 $$ {\mathbf{x}}\left( {\mathbf{t}} \right) = \int\nolimits_{{ - \infty }}^{{ + \infty }} {{\mathbf{X}}\left( {\mathbf{f}} \right){\mathbf{e}}^{{ - {\mathbf{j}}2{\mathbf{\pi ft}}}} {\mathbf{dt}}~\;{\mathbf{with}} - \infty < f < + \infty } $$

The most notable feature is the peak frequency (FPeak) within the PSD, which has been used extensively in heart rate variability studies [29]. It is regarded as a useful measure of variability and normal sympathetic and parasympathetic function. It describes the dominant frequency in the PSD that has the maximum spectral power. In this study, peak frequency is derived using Welch’s method [30]:

15 $$ {\text{FPeak}} = \hbox{max} \left( {\mathop \sum \limits_{i = 1}^{N} s_{i} \left( i \right)} \right) $$

where ${\text{s}}_{i} \left( i \right)$ is the power of the spectrum at bin *i*. As shown later in the paper, this feature has good discriminative capacity as a confounding coefficient.

#### Non-linear features

Poincare plots are a geometrical representation of a time series that is also used extensively to measure heart rate variability [20]. This paper shows that it has excellent discriminatory capacity in CTG analysis. Unlike HRV where it is commonly used, in FHR the difference between two beats is given as NN rather than the RR interval. A line of identity is used as a 45° imaginary diagonal line on the plot and the points falling on the line have the property $NN_{n} = NN_{n + 1}$ [31]. Three coefficients of the Poincare plot, SD1 (the standard deviation of points perpendicular to the axis of line-of-identity), SD2 (the standard deviation of points along the axis of line-of-identity) and SDRatio are used as features to describe the cloud of points in the plot. Fundamentally, SD1 and SD2 are directly related to the standard deviation of NN interval (SDNN) and the standard deviation of the successive difference of the NN interval (SDSD) that is given by:

16 $$ \begin{aligned} {\text{SD}}1^{2} = \frac{1}{2}{\text{SDSD}}^{2} = {\text{Y}}_{\text{NN}} \left( 0 \right) - {\text{Y}}_{\text{NN}} (1) \hfill \\ {\text{SD}}2^{2} = 2{\text{SDNN}}^{2} - \frac{1}{2}{\text{SDSD}}^{2} = {\text{Y}}_{\text{NN}} \left( 0 \right) \hfill \\ + \,Y_{NN} \left( 1 \right) - 2\overline{{NN^{2} }} \hfill \\ \end{aligned} $$

where $Y_{NN} \left( 0 \right)$ and $Y_{NN} \left( 1 \right)$ describe the autocorrelation function for lag-0 and lag-1 of the NN interval, respectively. The mean of NN intervals is $\overline{NN}$. Equation 16 shows that SD1 and SD2 measures are derived from the correlation and mean of the NN intervals time series with lag-0 and lag-1.

The SD1 feature is an index of instantaneous recording of the beat-to-beat short-term variability (the parasympathetic action) and SD2 describes the long-term variability (the sympathetic action). SD1 and SD2 are combined to form the ratio of SD1/SD2 that shows the relation between short and long-term variations of NN intervals:

17 $$ {\text{SDRatio}} = \pi \times {\text{SD}}_{1} \times {\text{SD}}_{2} $$

It is also possible to detect the existence of chaos in the FHR signal since the foetal heartbeat fluctuates on different time scales and has the property of being self-similar. In this study, the box-counting dimension is used to estimate the dynamics of the FHR [32]. It is a quantitative measure of the morphological properties of a signal and its capacity that is determined by covering the signal with *N* boxes of side length *r*. The minimal number of optimally sized boxes required to cover the complete signal describes the box-counting dimension coefficient such that:

18 $$ {\text{D = }}\mathop { \lim }\limits_{{{\text{r}} \to 0}} \frac{{{\text{logN}}\left( {\text{r}} \right)}}{{{ \log }\left( { 1 / {\text{r}}} \right)}} $$

where *D* is the box counting fractal dimension of the object, *r* is the side length of the box, and N(*r*) is the smallest number of boxes of side *r* to cover the time series signal.

Long-term time-correlations or self-affinity measures of the FHR signal have also proven in previous studies to be useful for separating normal and pathological cases [33]. In this study, detrend fluctuation analysis (DFA) is performed where the returned exponent value indicates the presence or absence of fractal properties, i.e. self-similarity. The DFA probes the signal at different time scales and provides a fractal scaling exponent *x*. First the times series is integrated as follows:

19 $$ {\text{y}}\left( {\text{k}} \right){ = }\sum\limits_{{\text{i}}= 1}^{\text{k}} {{\text{X}}\left( {\text{i}} \right) - {\text{X}}_{\text{avg}} } $$

where y(*k*) is the cumulative sum of the *i*th sample and X_avg_ is the mean value of the entire signal. Windows are derived from y(*k*) of equal length *n* and linear approximations $y_{n}$ are found using least squares fit (this represents a trend in a given window). The average fluctuation F(*n*) of the signal around the trend is given by:

20 $$ {\text{F}}\left( {\text{n}} \right) = \sqrt {\frac{ 1}{\text{N}}\sum\limits_{{\text{k}= 1}}^{\text{N}} {\left( {{\text{y}}({\text{k}}) - {\text{y}}_{\text{n}} ({\text{k}})} \right)^{2} } } $$

The calculations are repeated for all values of n. In this instance the primary focus is the relation between F(*n*) and the size of the window *n*. In general F(*n*) increases with the size of window *n*.

### Feature selection

Feature selection is performed using the recursive feature eliminator algorithm (RFE) [34]. In this study a feature set was derived from the raw FHR signals based on the feature definitions described and a model fit generated using the RFE algorithm (refer to Algorithm 1) [34].

Each feature within this set is ranked using its importance to the model where *S* is a sequence of ordered numbers, which are candidate values for the number of features to retain $(S_{1} > S_{2,  \ldots } )$. This process is repeated and the $S_{i}$ top ranked features are retained. The model is refit and the performance is reassessed. The top $S_{i}$ features are used to fit the final model.

### Synthetic minority oversampling technique

In a two class balanced dataset the prior probabilities will be equal for each. This is not the case for the CTU-UHB dataset given there are 506 controls (majority class) and 46 cases (minority class). Classifiers are more sensitive to detecting the majority class and less sensitive to the minority class and this leads to biased classification [35]. Therefore, given a random sample taken from the dataset, the probability of a classifier classifying a foetus observation as a control will be much higher (91.6%–506/552) than the probability of it classifying a foetus observation as a case (8.3%–46/552). This imposes a higher cost for misclassifying the minority (predicting that a foetus is normal and the outcome being pathological) than the majority class, (predicting a foetus is pathological and the outcome being normal).

In order to address this problem, it is necessary to resample the dataset [36]. Various resampling techniques are available, and these include under sampling and over sampling. Under sampling reduces the number of records from the majority class to make it equal to the minority class—in this instance it would mean removing 460 records leaving us with a very small dataset. In contrast, data in the minority class can be increased using oversampling. In this study, the synthetic minority over-sampling technique (SMOTE) as defined in Algorithm 2 is used rather than reducing the dataset further [37].

Several studies have shown that the SMOTE technique effectively solves the class skew problem [35, 38–42]. Using SMOTE the minority class (cases) is oversampled using each minority class record, in order to generate new synthetic records along line segments joining the *k* minority class nearest neighbours. This forces the decision region of the minority class to become more general and ensures that the classifier creates larger and less specific decision regions, rather than smaller specific ones. In  [37] the authors indicated that this approach is an accepted technique for solving problems related to unbalanced datasets.

### Machine learning classifiers

#### Deep learning classifier

Deep learning neural network architectures have recently proven to be very powerful classifiers [43]. To the best of our knowledge, this algorithm has not been used in CTG studies, and this paper is thus the first to consider its use in automated CTG analysis. A multi-layer feedforward neural network architecture is used based on theoretical proofs in [44]. The supervised training phase is based on uniform adaptive optimized initialization that is determined by the size of the network. A Tansigmoid nonlinear activation function *f* is utilized and defined as:

21 $$ \begin{aligned} f(\alpha ) = \frac{{e^{\alpha } - e^{ - \alpha } }}{{e^{\alpha } + e^{ - \alpha } }} \hfill \\ where\,f\left( \cdot \right) \in [ - 1,1] \hfill \\ and\,\alpha = \mathop \sum \limits_{i} w_{i} x_{i} + b \hfill \\ \end{aligned} $$

where $x_{i}$ and $w_{i}$ represent the firing neuron’s input values and their weights, respectively; and α denotes the weighted combination.

The multinomial distribution is adopted with the cross-entropy loss function, which is typically used for classification in deep learning. For each training example *j*, the objective is to minimize a loss function:

22 $$ L(W,B|j) $$

where *W* is the collection $\{ W_{i} \}_{1:N - 1}$, $W_{i}$ denotes the weight matrix connecting layers *i* and *i* + 1 for a network of *N* layers. Similarly *B* is the collection $\{ b_{i} \}_{1:N - 1}$, where $b_{i}$ denotes the column vector of biases for layer *i* + 1. In the case of cross entropy, the loss function can be calculated by:

23 $$ L\left( {W,B|j} \right) = - \mathop \sum \limits_{y \in O} \ln \left( {O_{y}^{\left( j \right)} } \right) \cdot t_{y}^{j} + \ln \;(1 - O_{y}^{j} ) \cdot (1 - t_{y}^{j} ) $$

where $t^{(j)}$ and $O^{(j)}$ are the predicted and actual outputs, respectively, training example *j*, *y* represents the output units, and *O* the output layer.

The process used in this study to minimize the loss function defined in (22) is stochastic gradient descent (SGD) (refer to Algorithm 3) [45].

To address the problem of overfitting the dropout regularization technique proposed in [45] is used. This ensures that during forward propagation, when a given training example is used, the activation of each neuron in the network is suppressed within probability *P*. This coefficient is typically <0.2 for input neurons and <=0.5 for hidden neurons. Dropout allows an exponentially large number of models to be averaged as an ensemble, which helps prevent overfitting and improve generalization.

Momentum and learning rate annealing are used to modify back-propagation to allow prior iterations to influence the current version. In particular a velocity vector, *v*, is defined to modify the updates:

24 $$ \begin{aligned} \upsilon_{t + 1} = \mu \upsilon_{t} - \alpha \nabla L\left( {\theta_{t} } \right) \hfill \\ \theta_{t} + 1 = \theta_{t} + \upsilon_{t + 1} \hfill \\ \end{aligned} $$

where $\theta$ describes the parameters *W* and *B*, $\mu$ the momentum coefficient, and $\alpha$ the learning rate. Using the momentum parameter helps to avoid local minima and any associated instability [46]. Learning rate annealing is used to gradually reduce the learning rate $\alpha_{t}$ to “freeze” into a local minima in the optimized landscape and is based on the principles described in [47].

#### Fishers linear discriminant analysis classifier

Before the more advanced random forest classification model is considered this section discusses the fishers linear discriminant analysis (FLDA) classifier as a baseline classification model. FLDA finds a linear combination of features that determines the direction along which the two classes are best separated. In this study the criterion proposed by Fisher is used which is the ratio of between-class to within-class variances. The data is projected onto a line, and the classification is performed in this one-dimensional space. The projection maximizes the distance between the means of the two classes while minimizing the variance within each class:

25 $$ {\text{f}}\left( {\text{y}} \right) = W^{T} X + \alpha $$

where $\alpha$ is the bias, *W* is calculated using Fishers LDA, and *X* is the training data without class labels such that $f\left( y \right) \ge 0$ for normal records and <0 for pathological records. *W* is derived from *X* such that the within class scatter matrix $S_{W}$ is minimized by:

26 $$ S_{W} = \mathop \sum \limits_{i = 1}^{C} \mathop \sum \limits_{{x_{k} \in X_{i} }} (x_{k} - \mu_{i} )(x_{k} - \mu_{i} )^{t} $$

where *C* is the number of classes, $X_{i}$ is the set of all points that belong to class *i*, $\mu_{i}$ is the mean of class *i*, and $X_{k}$ is the *k*th point of $X_{i}$. The between class scatter matrix $S_{B}$ is maximized by:

27 $$ S_{B} = \mathop \sum \limits_{i = 1}^{C} N_{i} (\mu_{i} - \mu )(\mu_{i} - \mu )^{t} $$

where *C* is the number of classes, $N_{i}$ is the total number of points that belong to class *i*, $\mu_{i}$ is the mean of class *i*, and $\mu$ is the overall mean, i.e. the mean of the data when all classes are considered together.

#### Random forest classifier

Random forest (RF) classifiers have featured widely in biomedical research [11, 48–50]. They are based on an ensemble of many randomized decision-trees that are used to vote on the classification outcome. Many studies have shown that they give classification accuracies comparable with the best current classifiers on many datasets. They are able to handle data with a large number of features. Those features that are important for classification are determined through the calculation of an importance score for each feature. Each decision-tree is randomized using a bootstrap statistical resampling technique, with random feature selection [51].

Given an *M* feature set, trees are constructed using *m* features randomly selected from the feature set at each node of the tree. The best split is calculated using these *m* features, which continues until the tree is fully grown without pruning. The procedure is repeated for all trees in the forest using different bootstrap samples of the data. Classifying new samples can then be achieved using a majority vote. The approach combines bagging with decision tree classifiers to achieve this (refer to Algorithm 4).

### Performance measures

k-fold cross validation is used as a prediction metric with fivefolds and 1 and 30 repetitions, respectively. The average performance obtained from 30 simulations is utilized. This number is considered, by statisticians, to be an adequate number of iterations to obtain an acceptable average. Let $C_{k}$ denote the indices of the observations in part *k*, and $n_{k}$ the number of observations in *k*: if *n* is a multiple of K, then $n_{k} = n/K$. Compute:

28 $$ CV_{k} = \mathop \sum \limits_{k - 1}^{k} n_{k} MSE_{k} $$

where

29 $$ MSE_{k} = \mathop \sum \limits_{{i \in C_{k} }} (x_{i} - \widehat{x}_{i} )^{2} /n_{k} $$

and $\widehat{x}_{i}$ is the fit for observation *i*, obtained from the data with part *k* removed.

Sensitivity (true positives) and specificity (true negatives) measure the predictive capabilities of classifiers in binary classification tests. Sensitivities refer to the true positive rate or recall rate (pathological cases). Specificities measure the proportion of true negatives (normal cases). Sensitivities are considered higher priority than specificities, in this study. It is important to predict a pathological case rather than miss-classify a normal case. To evaluate the performance of classifiers fitted to imbalanced datasets the F-Measure is a useful metric that combines precision and recall into a single value with equal weighting on both measures [52].

The area under the curve (AUC) is an accepted performance metric that provides a value equal to the probability that a classifier will rank a randomly chosen positive instance higher than a randomly chosen negative one (this obviously assumes that positive ranges higher than negative). This has been chosen, as it is a suitable evaluation method for binary classification. Consider a classifier that gives estimates according to $p(C_{i} |x)$, it is possible to obtain values $\{ a_{1} , \ldots , a_{n1} ; a_{i} = p\left( {C_{1} |x} \right), x_{i} \in C_{1} \}$ and $\{ b_{1} , \ldots , b_{n2} ; b_{i} = p\left( {C_{2} |x} \right), x_{i} \in C_{2} \}$ and use them to measure how well separated the distributions of $\widehat{p}(x)$ for class $C_{1}$ and $C_{2}$ patterns are [53].

Using the estimates, $\{ a_{1} , \ldots , a_{n1} ,b_{1} , \ldots , b_{n1} \}$ they can be ranked in increasing order. The class $C_{1}$ test points can be summed to see that the number of pairs of points, one from class $C_{1}$ and one from $C_{2}$ with $\widehat{p}(x)$ smaller for class $C_{2}$ than the $\widehat{p}(x)$ value for class $C_{1}$, is:

30 $$ \mathop \sum \limits_{i = 1}^{{n_{1} }} \left( {r_{i} - i} \right) = \mathop \sum \limits_{i = 1}^{{n_{1} }} r_{i} - \mathop \sum \limits_{i = 1}^{{n_{1} }} i = S_{0} - \frac{1}{2}n_{1} \;(n_{1} + 1) $$

where $r_{i}$ is the ranked estimate, $S_{o}$ is the sum of the ranks of the class $C_{1}$ test patterns. Since there are $n_{1} n_{2}$ pairs, the estimate of the probability that a randomly chosen class $C_{2}$ pattern has a lower estimated probability of belonging to class $C_{1}$ than a randomly chosen class $C_{1}$ is:

31 $$ \widehat{A} = \frac{1}{{n_{1} n_{2} }}\left\{ {S_{0} - \frac{1}{2}n_{1} (n_{1} + 1)} \right\} $$

This is equivalent to the area under the ROC which provides an estimate obtained using the rankings alone and not threshold values to calculate it [51].

## Results

This section presents the classification results for control and case records using the CTU-UHB dataset. The features extracted from the FHR signals are used to model each of the classifiers. The performance is measured using sensitivity, specificity, AUC, F-Measure and MSE values.

### Using all features from original data

In the first evaluation, all the features in the feature set are used to train the FLDA, RF and DL classifiers.

#### Classifier performance

The results in Table 2 show that the sensitivities for all classifiers are very low, while corresponding specificities are high. This is expected given that there are a limited number of case records from which the classifiers can learn a suitable fit. The F-Measure is a good metric when using imbalanced datasets and provides a better indication of classifier performance than sensitivity, specificity and AUC. As can be seen the F-Measure for the FLDA and RF are low with slightly better results produced by the DL (only slight better than chance).

**Table 2:** Using all features from original data

Classifier	Sensitivity	Specificity	AUC	F-Meas.
FLDA	0.0230	0.9931	0.6763	0.3245
RF	0.0223	0.9921	0.7725	0.3154
DL	0.0008	0.9990	0.8711	0.5220

It is clear that the models are capable of classifying control records but not case records. This is because there are 506 controls and only 46 cases from which the classifiers can learn, which is significantly lower. The AUC values are relatively low for the FLDA with slightly higher values for the RF and higher values again for the DL. Yet, the Sensitivities, which are considered more important in this study, are all low. Table 3 shows the error estimate for fivefold cross-validation using both 1 and 30 repetitions.

**Table 3:** Cross-validation results using original data

Classifier	Cross-validation fivefold 1 repetition	Cross-validation fivefold 30 repetitions
	Error	Error
FLDA	0.0954	0.0900
RF	0.0848	0.0830
DL	0.0803	0.0327

The errors are consistent with the expected MSE base-rate of 8.3% (46 pathological/552 FHR records) with the exception of the DL which produced an MSE = 3%.

#### Model selection

**Fig. 2:**
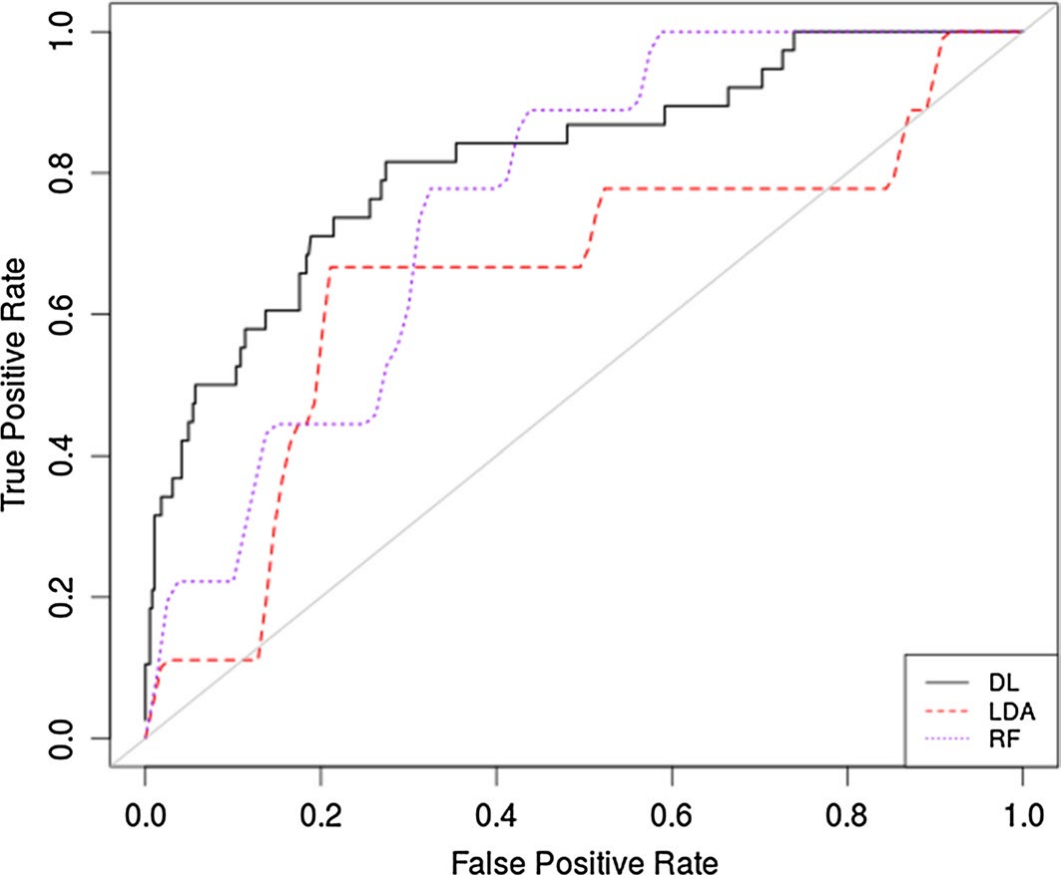
ROC *curve* for original data using all features

The receiver operator characteristic (ROC) curve is a useful graphic that shows the cut-off values for the false and true positive rates. It is particularly useful in binary classification to illustrate classifier performance. In the current evaluation, Fig. 2 shows that the FLDA performed poorly. The RF and DL classifiers produced slightly better results, which reflect the sensitivity, specificity and AUC values in Table 2.

The primary reason for the low sensitivities (despite the AUC for the RF and DL being relatively high) is that there are insufficient case records to model the class. This is in contrast to the classification of control records that are skewed in its favour. This causes significant problems in machine learning. As such, re-sampling the classes in the absence of real pathological cases is a conventional way of addressing this problem [54].

### Using all features from synthetic minority over-sampling technique data

**Fig. 3:**
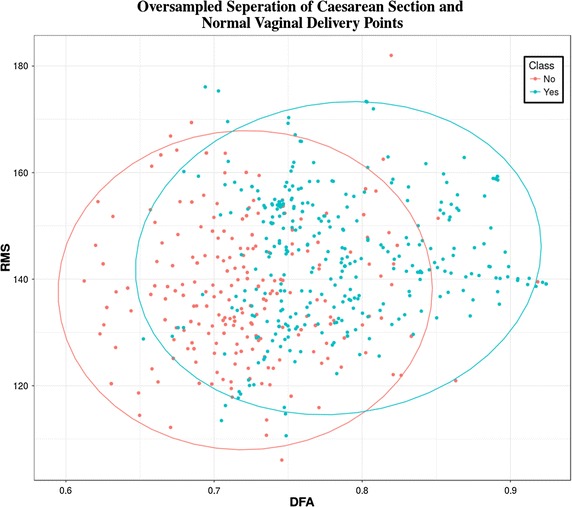
Oversampled separation of caesarean section and normal vaginal delivery points

The 46 case records are re-sampled using the SMOTE algorithm. The SMOTE algorithm generates a new dataset containing extra cases derived from the minority class while reducing the majority class samples accordingly. Figure 3 shows the separation of classes following oversampling. Compared with Fig. 1 it is clear that both case and control data are now evenly distributed between the two groups. There is significant overlap between case and controls and no two feature combinations were able to increase this decision boundary any further than that presented in Fig. 3.

#### Classifier performance

Using the new SMOTEd feature set (300 cases and 300 controls—empirically this distribution produced the best Sensitivity, Specificity, AUC, F-Measure and MSE results), Table 4 indicates that the Sensitivities for all models improved (90% in most cases). This is however at the expense of lower specificities (10% decreases). The results are encouraging given that accurately predicting cases is more important than predicting controls. The F-Measure acts as a support metric in this evaluation and produces encouraging results in the RF and DL classifiers.

**Table 4:** Using all features from SMOTE data

Classifier	Sensitivity	Specificity	AUC	F-Meas.
FLDA	0.6973	0.7875	0.7875	0.8128
RF	0.9291	0.9185	0.9812	0.9548
DL	0.9378	0.9099	0.9997	1.0000

Table 5 shows a marked improvement in error rates in all classifiers except the FLDA, which has increased by 12%. In the case of the DL classifier, the results indicate a 1.7% error rate, which is significantly less than the expected MSE base-rate of 50% (300 cases/600 FHR records).

**Table 5:** Cross-validation results using SMOTE data

Classifier	Cross-validation fivefold 1 repetition	Cross-validation fivefold 30 repetitions
	Error	Error
FLDA	0.2170	0.2315
RF	0.0940	0.1079
DL	0.0740	0.0168

#### Model selection

**Fig. 4:**
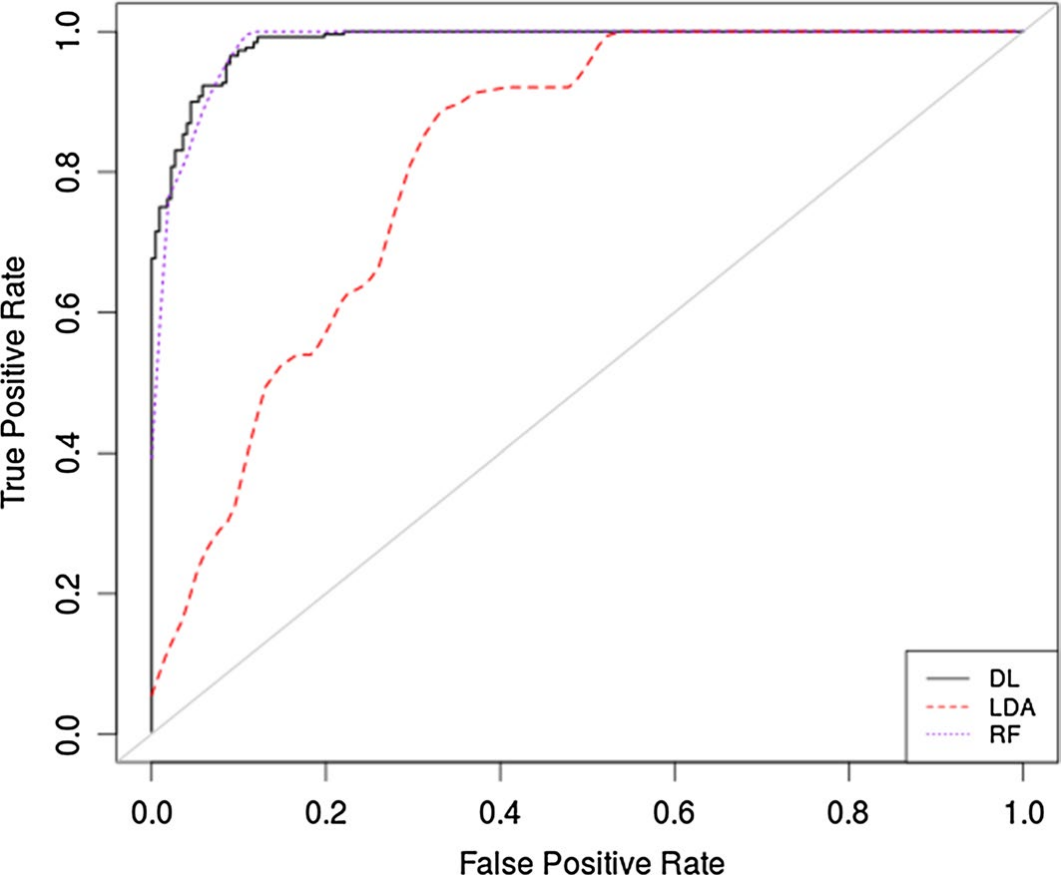
ROC *curve* for SMOTE oversampled data using all features

The ROC curve in Fig. 4 illustrates that all the models have significantly improved with the exception of the FLDA where the overall performance remained more or less the same.

The results show that adopting the SMOTE oversampling technique improves classifier performance. While oversampling data is not ideal, it is an accepted technique within many clinical studies when skewed datasets need to be normally distributed [54–56].

The remaining evaluations build on these results with a particular focus on dimensionality reduction.

### Using RFE selected features from SMOTE data

#### Recursive feature extraction (RFE)

**Fig. 5:**
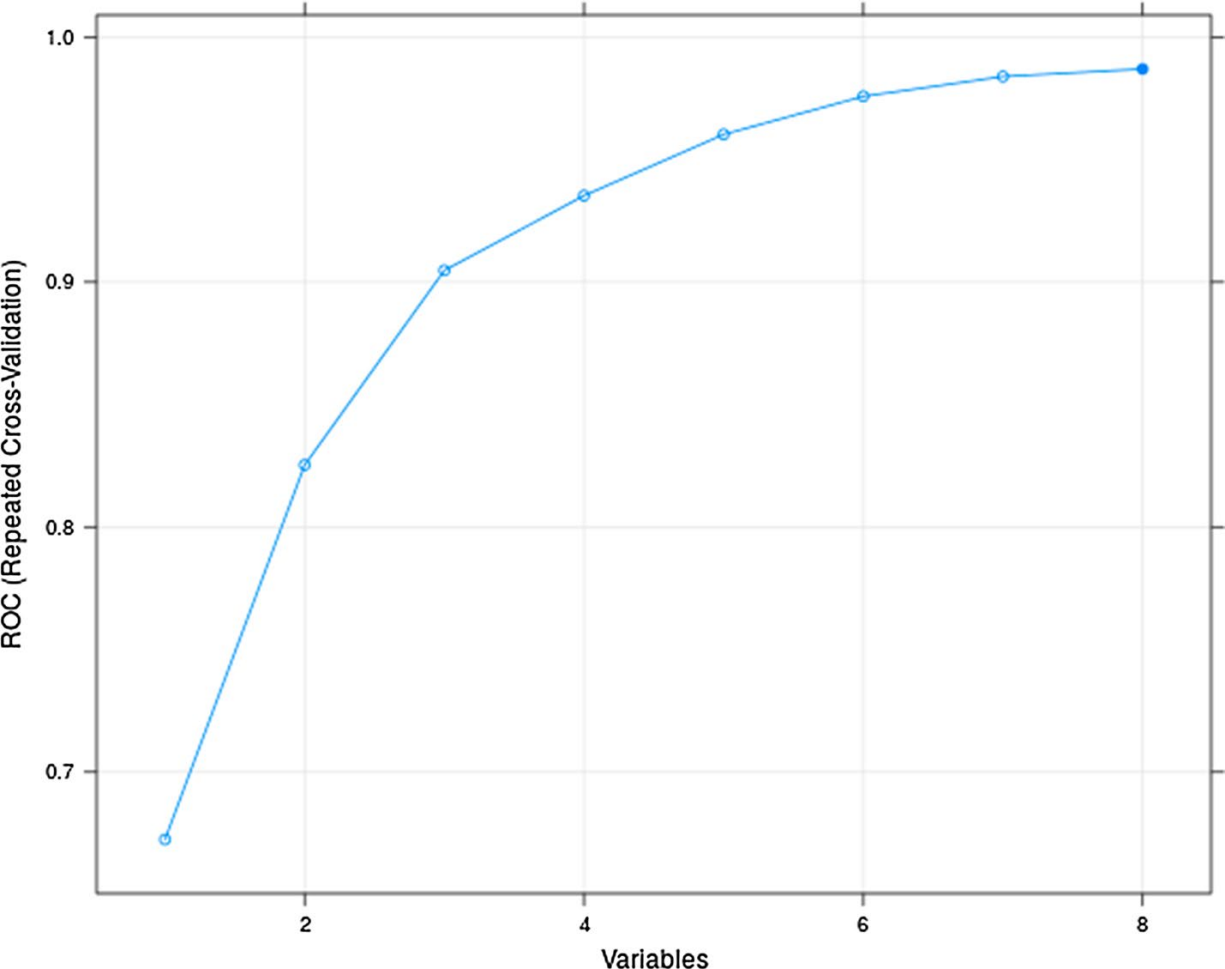
RFE feature ranking

Using the RFE algorithm, each feature is assessed to determine their discriminatory capacity. Figure 5 shows the cross-validation results using various feature combinations.

The results indicate that the optimal number of features is eight as can been seen in Table 6.

**Table 6:** RFE feature ranking

Variables	Sensitivity	Specificity	ROC
1	0.6644	0.6040	0.6724
2	0.7615	0.7422	0.8253
3	0.8119	0.8175	0.9047
4	0.8341	0.8817	0.9353
5	0.8393	0.9263	0.9603
6	0.8652	0.9409	0.9758
7	0.8644	0.9605	0.9839
8	0.8778	0.9675	0.9870

The eight ranked features are DFA, RMS, FPeak, Acc, SD2, SDRatio, SAMPEN, and Dec. The following evaluation determines whether this reduced feature set can improve on or maintain the previous set of results.

#### Classifier performance

Looking at Table 7 it can be seen that most of the classifiers perform slightly worse using the eight features in terms of sensitivity. This is with exception to the RF classifier, which can maintain similar results using the reduced feature set.

**Table 7:** Using RFE features from SMOTE data

Classifier	Sensitivity	Specificity	AUC	F-Meas.
FLDA	0.6169	0.7512	0.7564	0.7812
RF	0.9079	0.9135	0.9764	0.9138
DL	0.8314	0.8880	0.9980	1.0000

The MSE values, reported in Table 8, for all but the RF classifier (whose error more or less stayed the same) were slightly worse than in the previous evaluation.

**Table 8:** Cross-validation results using SMOTE data with RFE

Classifier	Cross-validation fivefold 1 repetition	Cross-validation fivefold 30 repetitions
	Error	Error
FLDA	0.2666	0.2719
RF	0.1068	0.1063
DL	0.0142	0.0343

#### Model selection

**Fig. 6:**
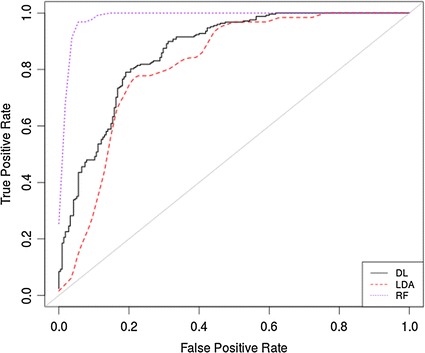
ROC curve for the SMOTE data using RFE features

In this final evaluation, the ROC curve in Fig. 6 illustrates that there are no real improvements on the previous evaluation for the FLDA and DL, but that the RF performs very well with a reduced set of features.

## Discussion

Obstetricians and midwives visually inspect CTG traces to monitor the wellbeing of the foetus during antenatal care. However, inter- and intra-observer variability and low positive prediction is accountable for the 3.6 million babies that die each year. This paper, presented a proof-of-concept using machine learning and FHR signals as an ambulatory decision support to antenatal care. The results indicate that it is possible to provide high predictive capacity when separating normal vaginal deliveries and caesarean section deliveries and in many cases produce much better results than those reported in previous studies (see Table 9).

**Table 9:** Comparison of previous works

Paper	Year	Classifier	Sensitivity	Specificity
[17]	2013	Naïve bayes	0.91	0.95
[6]	2014	RF and LCA	0.72	0.78
[57]	2013	LCR	0.66	0.89
[58]	2013	ANN	0.60	0.67
[20]	2012	SVM	0.73	0.76
[59]	2012	WFSS	0.92	0.88
[8]	2009	SI	0.90	0.75
[60]	2010	SVM	0.70	0.78

Using the original unbalanced dataset the best classifier (DL classifier) achieves SE = 0%, SP = 99%, AUC = 87%, and F-Measure = 52%. While the specificity values are high, all sensitivity values are below 3%. The low Sensitivity is attributed to the disproportionate number of normal records compared with pathological records and the fact that unbalanced datasets in general cause bias in favour of the majority class. The minimum error rate MSE = 3% was achieved by the DL using 30 repetitions. This relatively small MSE appeared to be a good error rate. However, the classifiers were simply classifying by minimizing the probability of error, in the absence of sufficient evidence to help them to classify otherwise.

The SMOTE algorithm using all 13 features significantly improved the Sensitivity values for all classifiers. While oversampling is not ideal, it is a way to solve the class skew problem that is widely used in medical data analysis [40, 61–64]. The best classification algorithm is again the DL classifier, which achieves SE = 94%, SP = 91%, AUC = 100%, F-Measure = 100% and MSE = 2%. The reason for this is that the algorithm has the ability to extract complex non-linear patterns generally observed in physiological data like FHR signals. Through the extraction of these patterns, the DL algorithm uses relatively simpler linear models for data analysis tasks, such as classification. The DL generalizes, and finds the global minima, which allows it to generate learning patterns and relationships beyond immediate neighbours in data. It is able to provide much more complex representations of data by extracting representations directly from unsupervised data without domain knowledge or inference.

Using the RFE algorithm as a feature selection technique the algorithm eliminated five features from the original 13 that were considered to have very low discriminatory capacity. The remaining eight features were used to fit the models and the results show that the RF achieved the best overall results with SE = 91%, SP = 91%, AUC = 98%, F-Measure = 91% and MSE = 11%. The primary reason for these good results is that the RF algorithm is based on an ensemble of many randomized decision-trees that are used to vote on the classification outcome. They are able to handle data with a very large number of features (although the feature set in this study is not particularly large) and those features that are important for classification can be determined through the calculation of an importance score for each feature. The score metric based on voting is similar to the approach adopted in *k*-nearest neighbour classification and the voting mechanism to classify new data points based on the majority surrounding data points of a particular class. The DL classifier performed worse on the reduced dataset but still produces better results than several studies discussed in this paper [6, 20, 57, 60].

## Conclusions and future work

The primary aim in this paper was to evaluate a proof-of-concept approach to separating caesarean section and normal vaginal deliveries using FHR signals and machine learning. The results show that using a deep learning classifier it is possible to achieve 94% for Sensitivity, 91% for Specificity, 99% for AUC, 100% for F-score, and 1% for Mean Square Error. This shows significant improvements over the 30% positive predictive value achieved by obstetricians and midwives and warrants further investigation as a potential decision support tool for use alongside the current CTG gold standard.

Nonetheless, despite the encouraging results reported, the study needs further evaluation using truly independent data to fully assess its value. In future work this will be made possible by soliciting support for clinical trials and utilising other open datasets that have adopted a similar study design. Other important work will include regression analysis, using a larger number of classes to predict the expected pathological event, in terms of the number of hours or days to delivery, not just whether the outcome is likely to be a caesarean section or a normal vaginal delivery. We also need to integrate and use the clinical data provided with this study in future analysis tasks.

It will also be important to evaluate different parameter adjustment settings, particularly in the case of the DL algorithm to determine if the results can be further improved. Automatic feature detection will also be explored using the DL to extract features from the raw FHR signals.

It is less than ideal to use oversampled data. Therefore, another direction for future work will explore opportunities to obtain data through funded clinical trials. This will also help provide a much more in-depth account of the value of machine learning and its perceived benefits on predicting caesarean section and normal vaginal deliveries.

While only the FHR signal is considered in this paper, since it provides direct information about the foetus’s state, it would be useful to combine this signal with the UC signal, which has been studied in previous work [64].

Overall, the proposed methodology is robust, contributes to the biomedical data analytics field, and provides new insights into the use of deep learning algorithms when analysing FHR traces that warrants further investigation.

## Abbreviations

CTG: cardiotocography
ST: ST segment connects the QRS complex and the T wave
UHB: University Hospital in Brno
CTU: Czech Technical University
CTU-UHB: Czech Technical University–University Hospital in Brno
STAN S21/S31: product name for CTG analysis
Avalon FM 40/50: foetal monitor
FHR: foetal heart rate
UC: uterine contraction
FIGO: international federation of gynecology and obstetrics
NICE: National Institute for Health and Care Excellence
VBM: virtual baseline
RBL: real baseline
STV: short term variability
LTV: long term variability
RMS: root mean squares
SampEn: sample entropy
FFT: fast fourier transform
PSD: power spectral density
FPeak: peak frequency
HRV: heart rate variability
SD: standard deviation
DFA: detrend fluctuation analysis
SVM: support vector machine
DL: deep learning
RFE: recursive feature eliminator
SMOTE: synthetic minority oversampling technique
SGD: stochastic gradient descent
FLDA: fishers linear discriminant analysis
RF: random forest
AUC: area under the curve
ROC: receiver operator curve
MSE: mean square errors

## Footnotes

1
http://www.cdc.gov/.

2
http://www.hscic.gov.uk.

3
http://www.figo.org/.

4
https://www.nice.org.uk/.
